# Depression, anxiety, and the COVID-19 pandemic: Severity of symptoms and associated factors among university students after the end of the movement lockdown

**DOI:** 10.1371/journal.pone.0252481

**Published:** 2021-05-27

**Authors:** Luke Sy-Cherng Woon, Mohammad Farris Iman Leong Bin Abdullah, Hatta Sidi, Nor Shuhada Mansor, Nik Ruzyanei Nik Jaafar

**Affiliations:** 1 Department of Psychiatry, Universiti Kebangsaan Malaysia Medical Centre, Cheras, Kuala Lumpur, Malaysia; 2 Lifestyle Science Cluster, Advanced Medical and Dental Institute, Universiti Sains Malaysia, Kepala Batas, Pulau Pinang, Malaysia; Maastricht University, NETHERLANDS

## Abstract

**Background and aims:**

This online cross-sectional study investigated the severity of depressive, anxiety, and stress symptoms among university students and determined the association between various factors and the levels of depressive and anxiety symptoms in response to the coronavirus disease 2019 (COVID-19) pandemic after the movement control order (MCO) was lifted.

**Methods:**

A total of 316 participants were administered a self-report questionnaire that collected data on sociodemographic attributes, personal characteristics, COVID-19-related stressors, religious coping, and clinical characteristics. In addition, the Multidimensional Scale of Perceived Social Support (MSPSS) and the 21-item Depression, Anxiety and Stress Scale (DASS-21) were administered.

**Results:**

Regarding depression, 15.5%, 11.7%, and 9.2% of the participants reported mild, moderate, and severe to extremely severe depression, respectively. For anxiety, 7.0%, 16.5%, and 13.2% of the respondents had mild, moderate, and severe to extremely severe anxiety, respectively. Moreover, 26.3% of participants had mild stress, 9.5% had moderate stress, and 6.6% had severe to extremely severe stress. The multiple linear regression model revealed that frustration because of loss of daily routine and study disruption and having preexisting medical, depressive, and anxiety disorders were associated with elevated depressive symptoms, while a greater degree of family and friends social support was associated with less depressive symptoms after adjusting for age, gender, and marital status. It was also found that frustration because of study disruption and having preexisting medical, depressive, and anxiety disorders were associated with elevated anxiety symptoms, while being enrolled in medicine-based courses and having a greater degree of family support were factors associated with less anxiety symptoms after adjusting for age, gender, and marital status.

**Conclusion:**

There is a need to conduct a longitudinal study in the future to confirm the causal relationship between the significant predictive factors and depression and anxiety identified in this study, and maintenance of a persistent flow of academic activities and social interaction may be of utmost importance to safeguard the mental wellbeing of university students.

## Introduction

The outbreak of coronavirus disease 2019, better known as COVID-19, took the world by surprise when it began to spread as a novel viral infection in China in December 2019. As a result of the rapid rise in the infection rate, a movement control order (MCO) was imposed in Malaysia from March 18 to June 10, 2020 [1]. Under the unprecedented rules introduced during the MCO, the Malaysian general population was confined to home activities. Any movement and social gatherings and religious, sports, social, or cultural activities, as well as educational activities (e.g., conducting physical classes and all activities involving public and private primary, secondary, and higher education institutions and training centers), were prohibited, while work-related activities were confined to working from home [2]. The MCO was followed by the recovery MCO (RMCO) between June 10 and August 31, 2020. Under the RMCO, some social activities were allowed under a strict standard operation procedure, but social distancing was still compulsory [3]. Nevertheless, academic activities continued to be limited; they were mostly offered on online platforms, and university students were not allowed physical access to the university’s facilities. All classes have been conducted electronically since April 2020.

Although there were similarities in the academic environment between the MCO and the time after the MCO was lifted, there were a few differences. During the MCO, lab-based medical sciences students were not allowed to enter the laboratory and continue with their research work, but after the MCO was lifted, lab-based students were allowed to continue their research work in the laboratory with social distancing. Moreover, during the MCO, students who were staying in the student hostels on and off campus when the MCO was declared were not allowed to go home to their family, but after the MCO was lifted, they could return home. In addition, there were several social activities that were less restricted after the MCO was lifted, such as going out for groceries, dining in restaurants and cafeterias, riding in cars with up to four persons, and religious activities, if the practice of social distancing was maintained.

During the movement lockdown, as a result of the disruption of academic and routine social activities, the prevalence of psychological sequelae, such as psychological stress, depression, anxiety, and acute stress reaction, increased among university students. Among the predisposing factors of poor mental health were sociodemographic characteristics (older age, female gender, having a non-medical major, living in rural areas or suburbs, and financial constraints), COVID-19-related stressors (remote online teaching, uncertainty about the future because of academic disruption, effects on daily life [e.g., decreased social interactions because of social distancing], disruption of sleep, limited recreational activities, having a relative or acquaintance who was COVID-19 positive, concern about own health and the health status of loved ones, fear of infection, and receiving negative information regarding the infection pandemic), psychological factors (history of childhood adversity and stressful life events, lower resilience, and higher anxious attachment), and a lifestyle factor (history of alcohol use). Protective factors against impaired mental health were sociodemographic variables (living in an urban area and having a stable family income) and psychosocial factors (living with family, good social support from peers, and good family functioning) [4–12]. Moreover, positive religious coping has been reported to be predictive of a lower severity of anxiety and depression in the Malaysian population [13].

Notwithstanding a few studies that investigated the psychological impact of COVID-19 on the Malaysian population, most of the studies on college students were conducted among university students outside Malaysia. There is a paucity of data on the mental health status of university students in relation to COVID-19 after the movement lockdown was lifted. Hence, this study aimed to fill the research gap via the following measures: (1) investigating the severity of depressive, anxiety, and stress symptoms in response to the COVID-19 pandemic after the movement lockdown was lifted among a cohort of Malaysian university students and (2) determining the association between the levels of depressive and anxiety symptoms on one hand and various COVID-19 stressors, religious coping, personal and clinical factors, and social support on the other. Our hypotheses were as follows: (i) Depressive, anxiety, and stress symptoms may have depreciated after the movement lockdown was lifted, but (ii) COVID-19-related stressors may predict elevated levels of depressive and anxiety symptoms, while social support may predict lower levels of depressive and anxiety symptoms, as found in existing literature.

## Materials and methods

### Sample

This cross-sectional online survey received approval from the Human Research Ethics Committee of Universiti Sains Malaysia (USM; USM/JEPeM/COVID19-21) and the Medical Research Committee of the Faculty of Medicine, Universiti Kebangsaan Malaysia (UKM; UKMPPI/111/8/JEP-2020-370). As described below, participants were recruited via snowball sampling in an email, which provided a link to an online survey system (Google Forms). The source population of the sample was all university students from the faculties of medicine of Malaysian public universities located in the Klang Valley, in the central region of Peninsular Malaysia, and the states of Penang and Kelantan, in the northern region of Peninsular Malaysia. The participants were recruited from three major public institutions, which were as follows: (1) the Advanced Medical and Dental Institute, USM, based in the state of Penang in northwest Peninsular Malaysia; (2) the School of Medical Sciences and School of Health Sciences, USM, based in the state of Kelantan in northeast Peninsular Malaysia; and (3) the Faculty of Medicine and Universiti Kebangsaan Malaysia Medical Centre, UKM, based in Klang Valley in central Peninsular Malaysia. USM has an estimated 28,300 students, while UKM has about 22,605 students from all the states in Malaysia.

Initially, a group of postgraduate students was invited to participate via email. The email contained information on the study purposes, procedures, and risks and benefits of participating in the study, as well as a link to the online survey system (Google Forms) whereby participants could respond to the questionnaires. They were then asked to extend the invitation to participate in the study by forwarding the invitation email to other postgraduate students and undergraduate students (medicine-based and medical science–based programs) within the faculties of medicine of public Malaysian universities in the targeted regions. This online survey was conducted from July 1 to July 21, 2020, which was approximately 3 weeks after the MCO was lifted by the Malaysian government. Those meeting the following criteria were eligible to participate in the study: (1) participants aged 18 years old and above with active student registration status in public Malaysian universities of the targeted regions in Klang Valley, Penang, and Kelantan; (2) no psychotic disorders or bipolar mood disorder; and (3) no history of illicit drug use. Informed consent for participating in the study was provided by the participants, who were assured of anonymity and the confidentiality of the information they disclosed. For those who had depression, anxiety, or psychological stress, the research team would recommend online mental health services (online counseling service for COVID-19 offered by USM) for these participants. Eventually, a total of 316 university students were recruited and enrolled in the study.

### Procedures

The detailed sociodemographic and personal characteristics, COVID-19-related stressors, religious coping, and clinical characteristics of the participants were assessed by a self-reported questionnaire. In addition, the Malay version of the Multidimensional Scale of Perceived Social Support (MSPSS) was administered to the participants to assess family, friends, and significant others social support, and the Malay version of the 21-item Depression, Anxiety and Stress Scale (DASS-21) was administered to assess the severity of depressive, anxiety, and stress symptoms.

Data on sociodemographic and personal characteristics, such as gender, age, marital status, religion, type of course enrolled in at university, monthly expenses, and living arrangements during the MCO, were recorded. The age of the participants was recorded as a continuous variable. The answer possibilities to gender were *male* and *female*. The response possibilities to marital status were *married* and *single or divorced*. While the response possibilities to religion were *non-Muslim* and *Muslim*. 63.1% of the population in Malaysia is Muslim. The religious distribution of the sample in our study was similar to the religious distribution in Malaysia, where 69.3% of our participants were Muslim, whereas only 30.7% of the participants were non-Muslim. Hence, we coded the responses to religion into Muslim and non-Muslim. The response choices for type of course enrolled in at university were *medical science–related courses (Bachelor of Science*, *Master of Science*, *or Doctorate)* and *medicine-related courses (Bachelor of Medicine and Surgery*, *Master of Medicine*, *or sub-specialization)*. While the answer possibilities to monthly expenses were *less than USD 243*, *USD 243 to 728*, and *more than USD 728*. Finally, the response choices for living arrangements during the MCO were *living alone*, *living with friends*, and *living with family*.

Data on COVID-19-related stressors, such as the presence of marital problems during COVID-19, concern about family during the MCO, duration of online classes attended per week, frustration because of loss of daily routine, frustration because of disruption of study, having fear when developing physical symptoms (e.g., fever, flu and cough), and history of quarantine because of exposure to COVID-19-positive cases, and the use of religious coping to manage stress during the COVID-19 pandemic, were also collected. The answer possibilities to the presence of marital problems during COVID-19, concern about family during the MCO, frustration because of loss of daily routine, frustration because of disruption of study, history of quarantine because of exposure to COVID-19-positive cases, and the use of religious coping to manage stress during COVID-19 were *no* and *yes*. The duration of online classes attended per week was recorded as a continuous variable, while the response possibilities to fear when developing physical symptoms (e.g., fever, flu, and cough) were *no*, *neutral*, and *yes*. Data on clinical characteristics included in the data collection were having preexisting medical and preexisting depressive and anxiety disorders, whereby the response possibilities were *no* and *yes*.

The MSPSS is a self-reported instrument used to assess the family, friends, and significant others social support received by the subjects. It consists of 12 items and three domains (family, friends, and significant others support), where each domain comprises four items. Each item is scored on a Likert scale ranging from 1 to 7. Hence, the range of the total scores can be from 12 to 84, while the cumulative score for each domain ranges from 4 to 28. A higher score indicates a greater degree of social support, while a lower score indicates less social support. The MSPSS has good internal consistency, with a Cronbach’s α of 0.88 [14]. The Malay version of the MSPSS has been validated among Malaysian university students and demonstrates excellent internal consistency, with a Cronbach’s α of 0.94 [15].

The DASS-21 is a self-administered tool used to assess the severity of depression, anxiety, and psychological stress symptoms. It comprises 21 items with three domains, where each domain (i.e., depression, anxiety, and psychological stress) is made up of seven items. Each item is scored on a Likert scale ranging from 0 to 3. The cumulative score of each subscale is calculated by summing up the scores of all its items and multiplying by two. Hence, the score of each subscale ranges from 0 to 42, and the total score ranges from 0 to 120. A higher (lower) score indicates greater (less) severity of depression, anxiety, or psychological stress. The cut-off scores for case findings in DASS-21 are as follows: 10 for the depression subscale, 7 for the anxiety subscale, and 11 for the stress subscale. The severity score ranges for depressive symptoms are as follows: mild depression = 10–12, moderate depression = 13–20, severe depression = 21–27, and extremely severe depression = 28–42. The severity score ranges for anxiety symptoms are as follows: mild anxiety = 7–9, moderate anxiety = 10–14, severe anxiety = 15–19, and extremely severe anxiety = 20–42. The severity score ranges for psychological stress are as follows: mild stress = 11–18, moderate stress = 19–26, severe stress = 27–34 and extremely severe stress = 35–42 [16]. The Malay version of the DASS-21 has acceptable internal consistency, with Cronbach’s α values of its subscales ranging from 0.74 to 0.79 [17].

### Statistical analysis

Data analyses were carried out with the Statistical Package for Social Sciences, version 26 (SPSS 26; SPSS Inc., Chicago, Illinois, USA). Descriptive statistics for sociodemographic and personal characteristics, stressors, clinical factors, mean scores of the MSPSS domains, and the severity and mean scores of depressive, anxiety, and stress symptoms were reported. There were no missing data. The frequency and percentage of participants in each group or category of all the categorical variables (marital status, gender, religion, type of course enrolled in at university, monthly expenses, living arrangements during the MCO, marital problems during COVID-19, concern about family during the MCO, frustration because of loss of daily routine, frustration because of disruption of study, fear when developing physical symptoms that resemble COVID-19 infection, history of quarantine because of exposure to COVID-19-positive cases, use of religious coping to manage stress during the COVID-19 pandemic, having preexisting medical illnesses and preexisting depressive and anxiety disorders) were reported. All continuous variables (age, depression, anxiety, stress, family support, friends support, and significant others support scores, as well as the hours of online class per week) were non-normally distributed, as indicated by the Shapiro–Wilk test (*p* < 0.05), histogram, and normal Q-Q plot. Hence, the continuous variables were reported as median and interquartile range (IQR).

The associations between various factors and the levels of depressive and anxiety symptoms were investigated. Multiple linear regression analyses were performed with COVID-19-related stressors; religious coping; clinical factors; and the degree of family, friends, and significant others social support as independent variables and the depressive and anxiety scores as dependent variables. Confounding sociodemographic variables, such as age, gender, and marital status, were adjusted for in the analyses. As a result of the non-normal distribution of the depressive and anxiety symptoms scores, bootstrapping with 2,000 replications was carried out for the two multiple linear regression models. Multicollinearity between independent variables in the multiple linear regression models was checked with the variance inflation factor (VIF); all the independent variables recorded VIF values of less than 5, indicating absence of multicollinearity. Statistical significance was set at *p* < 0.05 for the multiple linear regression analyses, and all *p*-values were two-sided.

## Results

### Participant characteristics

The sociodemographic and personal characteristics, COVID-19-related stressors, religious coping, and clinical factors of the participants are summarized in Table 1. The median age of the participants was 31 years (IQR = 11). Most of the participants lived with their family during the MCO. Among the COVID-19-related stressors, the commonest stressor was frustration because of study disruption during COVID-19, leading to feelings of uncertainty about the future as a consequence of delay in students’ graduation time, lack of practical sessions and guidance, difficulties in following online classes because of internet problems, difficulty adjusting to new norms of learning, and loss of momentum to study, in more than half of the participants. Frustration because of loss of daily routine during COVID-19 (e.g., loss of daily leisure and social activities and loss of daily academic routines, which included classes and clinical sessions) and the experience of tremendous fear when they developed physical symptoms (e.g., fever, flu, and cough) because of the perception that they had contracted COVID-19, affected almost half of the participants. Otherwise, the other COVID-19-related stressors, such as the presence of marital problems during COVID-19, concern about family during the MCO, and history of quarantine because of exposure to COVID-19-positive cases, were experienced by only a minority of participants. In addition, more than half of the participants believed that religion helped them to cope with the MCO and COVID-19, while only a small proportion of participants had preexisting medical illnesses or depressive and anxiety disorders.

**Table 1:** Sociodemographic and personal characteristics, COVID-19-related stressors and coping, and clinical factors of the participants.

Variable	*N*	%
**Age:**	31.0^a^	11.0^b^
**Gender:**		
Male	95	30.1
Female	221	69.9
**Marital status:**		
Married	126	39.9
Single/divorced	190	60.1
**Marital problems during COVID-19:**		
No	296	93.7
Yes	20	6.3
**Religion:**		
Non-Muslim	97	30.7
Muslim	219	69.3
**Did religion help you cope during the MCO and COVID-19?**		
No	101	32.0
Yes	215	68.0
**Type of course taken:**		
Medical science related (BSc/MSc/PhD)	69	21.8
Medicine related		
(MBBS/MMed/subspeciality)	247	78.2
**Monthly expenses:**		
< USD 243	115	36.4
USD 243 to 728	81	25.6
> USD 728	120	38.0
**Living arrangement during the MCO:**		
Living alone	27	8.5
Living with friends/course mates	23	7.3
Living with family	266	84.2
**Were you worried about your family during the MCO?**		
No	269	85.1
Yes	47	14.9
**Were you frustrated because of loss of daily routine during the MCO?**		
No	177	56.0
Yes	139	44.0
**Did you feel stress because your study was disrupted during the MCO?**		
No	107	33.9
Yes	209	66.1
**Median hours of online class per week during the MCO**	4.0^a^	4.0^b^
**Preexisting medical illnesses?**		
No	261	82.6
Yes	55	17.4
**Preexisting depressive and anxiety disorders?**		
No	301	95.3
Yes	15	4.7
**Were you afraid that you may have COVID-19 if you developed cough, flu, or fever?**		
No	49	15.5
Neutral	130	41.1
Yes	137	43.4
**Were COVID-19-positive cases prevalent in your area of living during the MCO?**		
No	222	70.3
Yes	94	29.7
**Quarantine for being exposed to COVID-19 patients:**		
No	299	94.6
Yes	17	5.4

^a^ = Median

^b^ = interquartile range (IQR)

The social support and psychological characteristics of the participants are illustrated in Table 2. The prevalence rates of depression, anxiety, and stress among the participants were 36.4%, 36.7%, and 42.4%, respectively (above the cut-off values for depression, anxiety, and stress in DASS-21). Regarding the severity of depressive symptoms, 15.5% of participants had mild depression (*n* = 49), whereas 11.7% had moderate depression (*n* = 37), and 9.2% had severe to extremely severe depression (*n* = 29). As for the severity of anxiety symptoms, only 7.0% of participants had mild anxiety (*n* = 22), whereas 16.5% of participants had moderate anxiety (*n* = 52), and 13.2% had severe to extremely severe anxiety (*n* = 42). Assessment of the severity of stress symptoms revealed that 26.3% of participants had mild stress (*n* = 83), whereas 9.5% of participants had moderate stress (*n* = 30), and 6.6% had severe to extremely severe stress (*n* = 21). Regarding social support, the medians of the family, friends, and significant others support scores were almost equal.

**Table 2:** Social support and psychological characteristics of the participants.

Variable	*n*	%
**Social support:**		
**Median family support score**	23.2^a^	6.6^b^
**Median friends support score**	22.0^a^	6.0^b^
**Median significant others support score**	23.2^a^	8.0^b^
**Depression:**		
Below cut-off score of 10	201	63.6
Cut-off score of ≥ 10	115	36.4
**Depression severity:**		
Below cut-off score of 10	201	63.6
Mild (score of 10–12)	49	15.5
Moderate (score of 13–20)	37	11.7
Severe (score of 21–27)	16	5.1
Extremely severe (score of ≥ 28)	13	4.1
**Median DASS-21 depression subscale score**	6.0^a^	10.0^b^
**Anxiety:**		
Below cut-off score of 7	200	63.3
Score of ≥ 7	116	36.7
**Anxiety severity:**		
Below cut-off score of 7	200	63.3
Mild (score of 7–9)	22	7.0
Moderate (score of 10–14)	52	16.5
Severe (score of 15–19)	22	7.0
Extremely severe (score of ≥ 20)	20	6.2
**Median DASS-21 anxiety subscale score**	4.0^a^	8.0^b^
**Stress:**		
Below cut-off score of 11	182	57.6
Score of ≥ 11	134	42.4
**Stress severity:**		
Below cut-off score of 11	182	57.6
Mild (score of 11–18)	83	26.3
Moderate (score of 19–26)	30	9.5
Severe (score of 27–34)	17	5.4
Extremely severe (score of ≥ 35)	4	1.2
**Median DASS-21 stress subscale score**	10.0^a^	10.0^b^

^a^ = Median

^b^ = interquartile range (IQR)

### The association between various COVID-19 stressors, religious coping, personal and clinical factors, and social support on one hand and the level of depressive symptoms on the other hand

Table 3 summarizes the multiple linear regression model (with bootstrapping with 2,000 replications) between various COVID-19 stressors, religious coping, personal and clinical factors, and social support on one hand and the level of depressive symptoms on the other, adjusted for confounding sociodemographic variables (e.g., age, gender, and marital status). The multiple linear regression model revealed that two COVID-19-related stressors, frustration because of loss of daily routine (*B* = 2.161, 95% CI = 0.414 to 3.777, *p* = 0.010) and frustration because of study disruption (*B* = 1.698, 95% CI = 0.296 to 3.007, *p* = 0.020), as well as two clinical factors—having preexisting medical illness (*B* = 3.110, 95% CI = 0.943 to 5.353, *p* = 0.017) and having preexisting depressive and anxiety disorders (*B* = 8.853, 95% CI = 3.411 to 13.937, *p* = 0.002)—were significantly associated with worsening of depressive symptoms. In contrast, greater family (*B* = -0.389, 95% CI = -0.687 to -0.084, *p* = 0.015) and friends support (*B* = -0.447, 95% CI = -0.704 to -0.153, *p* = 0.002) were protective against depressive symptoms.

**Table 3:** Association between various COVID-19 stressors, religious coping, personal and clinical factors, and social support on one hand and the level of depressive symptoms on the other among the participants.

Variable	*B* (95% CI)^a^	Standard error	*p*-value
**Marital problems during COVID-19:**			
No	Reference		
Yes	-1.051 (-3.970 to 1.974)	1.540	0.516
**Did religion help you cope with stress during the MCO and COVID-19?**			
No	Reference		
Yes	-0.882 (-2.703 to 0.653)	0.859	0.317
**Type of course enrolled in at university:**			
Medical science based	Reference		
Medicine based	-1.449 (-3.332 to 0.364)	0.923	0.120
**Living expenses per month:**			
≤ USD 728	Reference		
> USD 728	0.518 (-1.328 to 2.174)	0.869	0.546
**Living arrangement during the MCO:**			
Living alone/with friends	Reference		
Living with family	-3.123 (-11.185 to 5.447)	4.054	0.389
**Were you worried about your family during the MCO?**			
No	Reference		
Yes	-2.303 (-10.771 to 7.291)	4.188	0.551
**Frustration because of loss of daily routine:**			
No	Reference		
Yes	2.161 (0.414 to 3.777)	0.867	0.010*
**Hours of online classes attended per week**	0.019 (-0.144 to 0.143)	0.069	0.768
**Frustration because of study disruption:**			
No	Reference		
Yes	1.698 (0.296 to 3.007)	0.680	0.020*
**Preexisting medical illnesses:**			
No	Reference		
Yes	3.110 (0.943 to 5.353)	1.181	0.017*
**Preexisting depressive and anxiety disorders:**			
No	Reference		
Yes	8.853 (3.411 to 13.937)	2.649	0.002*
**Were you afraid if you developed cough, flu, or fever?**			
No/neutral	Reference		
Yes	0.015 (-1.475 to 1.611)	0.767	0.973
**Were COVID-19-positive cases prevalent in your place of living during the MCO?**			
No	Reference		
Yes	0.135 (-1.454 to 1.645)	0.784	0.843
**Quarantine because of exposure to COVID-19 patients:**			
No	Reference		
Yes	1.267 (-2.287 to 4.547)	1.785	0.509
**Family support score**	-0.389 (-0.687 to -0.084)	0.153	0.015*
**Friends support score**	-0.447 (-0.704 to -0.153)	0.145	0.002*
**Significant others support score**	-0.101 (-0.395 to -0.031)	0.095	0.087

* = Statistical significance at *p* < 0.05

^a^ = multiple linear regression model with bootstrapping with 2,000 replications reported that *F*(20,295) = 11.294, *p* < 0.001 with *R*^2^ = 0.434, adjusted for confounding sociodemographic variables (e.g., age, gender, and marital status)

### The association between various COVID-19 stressors, religious coping, personal and clinical factors, and social support on one hand and the level of anxiety symptoms on the other hand

Table 4 presents the multiple linear regression model (with bootstrapping with 2,000 replications) between various COVID-19 stressors, religious coping, personal and clinical factors, and social support on one hand and the level of anxiety symptoms on the other, adjusted for confounding sociodemographic variables (e.g., age, gender, and marital status). The multiple linear regression model reported that only one COVID-19-related stressor, such as frustration because of study disruption (*B* = 1.902, 95% CI = 0.163 to 3.246, *p* = 0.022), and two clinical factors—having preexisting medical illness (*B* = 3.803, 95% CI = 1.137 to 6.054, *p* = 0.002) and having preexisting depressive and anxiety disorders (*B* = 12.029, 95% CI = 4.346 to 20.614, *p* = 0.007); were significantly associated with worsening of anxiety symptoms. In contrast, students who were in medicine-based courses (*B* = -2.566, 95% CI = -4.607 to -0.561, *p* = 0.017) and a greater degree of family support (*B* = -0.292, 95% CI = -0.568 to -0.011, *p* = 0.035) exhibited a significant association with lower levels of anxiety symptoms.

**Table 4:** Association between various COVID-19 stressors, religious coping, personal and clinical factors, and social support on one hand and the level of anxiety symptoms on the other among the participants.

Variable	*B* (95% CI)^a^	Standard error	*p*-value
**Marital problems during COVID-19:**			
No	Reference		
Yes	-0.796 (-4.641 to 3.380)	1.978	0.718
**Did religion help you cope with stress during the MCO and COVID-19?**			
No	Reference		
Yes	-0.961 (-2.583 to 0.639)	0.848	0.272
**Type of course enrolled in at university:**			
Medical science based	Reference		
Medicine based	-2.566 (-4.607 to -0.561)	1.008	0.017*
**Living expenses per month:**			
≤ USD 728	Reference		
> USD 728	0.952 (-0.840 to 2.808)	0.913	0.332
**Living arrangement during the MCO:**			
Living alone/with friends	Reference		
Living with family	-2.984 (-10.011 to 4.068)	3.445	0.359
**Were you worried about your family during the MCO?**			
No	Reference		
Yes	-1.928 (-9.139 to 5.285)	3.400	0.554
**Frustration because of loss of daily routine:**			
No	Reference		
Yes	0.039 (-1.808 to 1.666)	0.851	0.973
**Hours of online classes attended per week**	0.075 (-0.098 to 0.230)	0.082	0.352
**Frustration because of study disruption:**			
No	Reference		
Yes	1.902 (0.163 to 3.246)	0.788	0.022*
**Preexisting medical illnesses:**			
No	Reference		
Yes	3.803 (1.137 to 6.054)	1.182	0.002*
**Preexisting depressive and anxiety disorders:**			
No	Reference		
Yes	12.029 (4.346 to 20.614)	4.148	0.007*
**Were you afraid if you developed cough, flu, or fever?**			
No/neutral	Reference		
Yes	0.448 (-1.078 to 2.186)	0.835	0.584
**Were COVID-19-positive cases prevalent in your place of living during the MCO?**			
No	Reference		
Yes	-0.637 (-2.181 to 1.043)	0.839	0.496
**Quarantine because of exposure to COVID-19 patients:**			
No	Reference		
Yes	2.971 (-1.630 to 8.323)	2.392	0.209
**Family support score**	-0.292 (-0.568 to -0.011)	0.131	0.035*
**Friends support score**	-0.119 (-0.337 to 0.137)	0.123	0.337
**Significant others support score**	-0.074 (-0.384 to 0.028)	0.091	0.130

* = Statistical significance at p < 0.05

^a^ = multiple linear regression model with bootstrapping with 2,000 replications reported that *F*(20,295) = 7.159, *p* < 0.001 with *R*^2^ = 0.327, adjusted for confounding sociodemographic variables (e.g., age, gender, and marital status)

## Discussion

This study investigated the severity of depressive, anxiety, and stress symptoms among Malaysian university students in response to the COVID-19 pandemic after the movement lockdown was lifted and determined the association between COVID-19-related stressors and religious coping, clinical factors, social support, and the levels of depression and anxiety symptoms. When we compared the levels of severity of depression, anxiety, and stress among the participants in our study with the results of a study on a cohort of Malaysian university students during the MCO, which used the same screening instrument (DASS-21; mean depression score = 4.61, anxiety = 4.28, and stress = 5.34) [18], the level of psychological symptoms in our study was proportionately higher. However, when we compared the rate of occurrence of depression, anxiety, and stress symptoms in our cohort of university students with that of another cohort of Malaysian university students in a non-pandemic setting with a relatively lower sample size (*n* = 91), which used the same screening instrument (DASS-21; mild depression = 14.3%, moderate depression = 19.8%, severe depression = 8.8%, extremely severe depression = 2.2%; mild anxiety = 25.3%, moderate anxiety = 28.7%, severe anxiety = 11.0%, extremely severe anxiety = 11.0%; mild stress = 19.8%, moderate stress = 11.0%, severe stress = 3.3%, extremely severe stress = 0%) [19], the rate of depression was comparable, the rate of anxiety was proportionately lower, and the rate of stress was proportionately higher in our study. The rate of occurrence of anxiety symptoms in other studies of Malaysian university students during the COVID-19 pandemic varied from 29.8% to 87.7% [12, 20, 21]; these discrepancies in the occurrence of anxiety symptoms between our study and other studies of Malaysian university students may be due to methodological differences and different screening tools used. (Our study utilized DASS-21, while other studies used Zung’s Self-Rating Anxiety Scale and the 7-item Generalized Anxiety Disorder Scale). Hence, we could not offer a conclusion for hypothesis (i) because there were only a few Malaysian studies for comparison, and they exhibited methodological differences and used different screening tools.

Our study findings confirmed hypothesis (ii) that COVID-related stressors (e.g., frustration because of loss of daily routine or study disruption) and clinical factors (e.g., having preexisting medical, depressive, and anxiety disorders) were associated with an increased level of depression. Conversely, greater social support (e.g., family and friends support) was protective against depression. It is not surprising that the loss of daily routine and study disruption resulting from social distancing as a preventive measure to control COVID-19’s spread could increase the risk of depression in university students, as has been reported in other studies [8, 22]. Isolation from social networks, loss of physical interaction, reduced social interaction, and less emotional support from others contributed to the higher risk of developing depression among university students [7, 8]. Other recognized factors include loss of recreational activities with family, friends, and significant others as a result of social distancing, which may also contribute to a higher risk of developing depression among university students [7, 8].

Like the findings reported in the general population in response to the COVID-19 pandemic, our study further confirmed that a history of medical illness, poor physical health, and a history of psychiatric illness were associated with worsening of depressive symptoms [23–25]. The reciprocal relationship between pre-existing medical illness and increased risk of depression can be understood in terms of the persistent fear of easily contracting the COVID-19 infection and the increased risk of mortality associated with chronic illness, which may lead to chronic stress and eventually increase the risk of depression [26]. Similarly, for those with psychiatric illness, they were constantly worried about their physical health. Persistent fear that they might have contracted the deadly infection when facing an infection pandemic and the poor coping strategies that followed increased their risk of depression [23].

Greater family and friends social support may have alleviated depression among the participants. Such social support may lead to an increase in the social network and enhance integration of the individual into the new social network. This may create increased resilience and enhance the likelihood of employing an active coping strategy in response to the COVID-19 pandemic, which can subsequently alleviate depression [27–29].

Consistent with hypothesis (ii) of our study, COVID-19-related stressor (e.g., frustration because of study disruption) was associated with increased anxiety symptoms. Similarly, clinical factors (e.g., having preexisting medical, depressive, and anxiety disorders) were also associated with worsening anxiety symptoms. The disruption of the study and academic progress of university students that occurred at times during the COVID-19 pandemic may have generated uncertainty about their future regarding their careers and academic achievement, which has been shown to increase the development of anxiety [4, 12]. The reasons for the frustration with study disruption among the university students in this study revealed that uncertainty about their future stemmed from delay in academic progress and postponement of the academic semester resulting from the COVID-19 pandemic and the MCO that followed. This led to a loss of momentum in their study, increasing their anxiety.

A history of psychiatric illness and the presence of chronic illness were reported as predictive factors of worsening anxiety symptoms in studies of the mental health status of the general population in the wake of the COVID-19 pandemic [30]. Similar findings were reported in our study, indicating that the constant fear of susceptibility to contracting COVID-19 infection because of the presence of chronic illness and exacerbation of anxiety symptoms in response to the pandemic in those who already had depressive and anxiety disorders may explain the elevation of anxiety symptoms in those with chronic and psychiatric illnesses.

Interestingly, we found that those enrolled in medicine-related courses (e.g., undergraduate medical students, doctors pursuing specialization courses and specialist trainees completing sub-specialization training) registered lower severity of anxiety symptoms. This could be because the experience of working as frontline staff during the COVID-19 pandemic among students enrolled in medicine-related courses increased their confidence in dealing with the pandemic, reducing their anxiety symptoms [31–34]. Higher social support and social support–seeking behavior have been shown to protect against development of anxiety symptoms among university students during the COVID-19 pandemic [20, 35]. The pivotal role of family support in this study could be comprehended by the finding that most of the university students in this study lived with their family (84.2%) during the MCO and throughout the COVID-19 pandemic. After the emergence of COVID-19, concern regarding family increased, while concern regarding leisure and friends decreased; people became more caring toward family and shared their feelings with family members [36, 37]. Hence, this facilitated an increase in perceived family support, which lowered the severity of anxiety symptoms among the participants in this study.

There were a few limitations in this study that should be considered. First, the cross-sectional design of this study did not allow the causal relationship between the various factors and depression and anxiety to be determined. Second, this study did not assess the role of personality traits in depression and anxiety symptoms, although they may act as confounding factors and affect the findings of the study [38, 39]. Third, to abide by the new norm of social distancing imposed by the government, this study utilized an online survey design and self-reported questionnaires, which may have led to response bias among the participants. Fourth, the responses for the variables of concerns about family, frustration because of loss of daily routine, frustration because of disruption of study, and fear of physical symptoms were coded as *yes* or *no* instead of being treated as continuous variables and the determination of the responses were based on the subjective account of the participants rather than specific criteria. This may have led to response bias. Finally, there were obvious imbalances in gender (only 30.1% of the participants were men) and religion distribution (69.3% of participants were Muslims) among the sample in our study, which may have affected the representativeness of our sample. Nevertheless, according to the national record of gender distribution among students in public higher education institutions in Malaysia in 2019, male student enrolment made up only 41.3% of the total number of students [40]. Hence, there was an imbalance in gender distribution among Malaysian university students at that time, where male students were much lower in proportion, and this imbalance is likely to have continued to the present. In addition, 63.1% of the population in Malaysia is Muslim. The religious distribution of our sample in this study was similar to the distribution for the country, where 69.3% of our participants were Muslims.

Despite its limitations, our study gave some new insights regarding the psychological impact of the COVID-19 pandemic among university students. First, there were a few COVID-19 related stressors that have not been evaluated in other studies of the psychological impact of COVID-19 among university students [4–12, 18, 20, 21, 34], such as the perception that religion helped to cope with stress during COVID-19, history of being in quarantine because of exposure to COVID-19-positive cases, and the perception that COVID-19-positive cases were highly prevalent around the area of residence. However, these factors turned out not to be associated with the levels of depressive and anxiety symptoms in our study. Second, clinical factors, such as having pre-existing medical illnesses, depressive, and anxiety disorders, were not assessed in other studies of psychological impact of COVID-19 among university students [4–12, 18, 20, 21, 34]. Our findings indicated that these two clinical factors were predictive of higher severity of depressive and anxiety symptoms among university students during COVID-19. Third, other studies of psychological impact of COVID-19 among university students [4–12, 18, 20, 21, 34] did not focus on which source of perceived social support was associated with severity of depressive and anxiety symptoms. This study revealed that higher family and friends social support predicted lower severity of depressive and anxiety symptoms among university students during the COVID-19 pandemic, but higher perceived social support from significant others did not have the same effect.

Based on our findings, we can suggest a few points to improve the mental health of university students (the stakeholders of higher education) and safeguard the quality of higher education as the COVID-19 pandemic continues. First, the higher education authority should arrange for and encourage the use of online social apps for education, demonstration, and discussion purposes for more interactive online classes and practical or clinical sessions. Novel effective methods of online student assessments to replace the old method of writing an examination or practical in-person assessment are necessary to avoid any delays in the academic progress of university students while ensuring the new norm of social distancing is practiced. This will help curb the uncertainty and loss of academic routine related to disruptions of daily routine and study, ultimately lowering the risk of developing depression and anxiety. In addition, a possible solution to curb the uncertainty about the future of university students which stemmed from delay in academic progress and postponement of the academic semester resulting from the COVID-19 pandemic and the MCO that followed is by proper rescheduling of classes and tutorials to provide ample time for students to compensate for the loss of momentum in their study. Extra tutorials to focus on “difficult-to-follow” topics should be arranged and specific academic areas which required in-person demonstration and practicum could be scheduled to enhance the learning outcomes of students, as now more countries are lifting restrictions related to campus closure. Institution of higher learning may also encourage and facilitate students to organize student support group and academic discussion group to enable students to discuss on personal matters which may disrupt their academic progress and academic topics which they may have difficulty to comprehend. Alternatively, a mentor-mentee group discussion consists of individual university lecturer as mentor and small group of students as mentee, may also benefit students who have personal matters which may disrupt their academic progress, particularly at this time where restrictions related to campus closure are being lifted, whereby in-person small group discussion is feasible. Finally, if there is a need to continue online classes and tutorials, the local communities and individual institution of higher learning may help by providing free internet access to university students living in the vicinity of the university. Poor internet access may hamper student attendance of online classes and tutorials.

Second, the education authority should pay more attention to students with medical, depressive, and anxiety disorders. Steps and standard operating procedures to ensure that these groups of students are on persistent treatment follow-up and adherence to medication should be implemented. The university authority may create online apps that allow the group of students with preexisting illness to record their clinic appointments and set reminders for their appointment dates. Telemedicine may also be useful where consultations and follow-up of students with preexisting medical and psychiatric illness could be carried out online. In fact, telemedicine has been reported to be effective for a wide range of medical and psychiatric illnesses, as well as increasing the efficacy of health services and demonstrating excellent technical effectiveness [41]. Because the group of students with preexisting medical, depressive, and anxiety disorders may have poor coping skills and adjustment in the face of a global pandemic, university counseling services and mental healthcare facilities should be made easily accessible online. Mindfulness and relaxation techniques, which are available for online learning, should be made available via university websites [42]. This step will help to curb depression and anxiety among university students with preexisting medical, depressive and anxiety disorders, and safeguard their mental wellbeing.

Finally, as family and friends social support are vital protective factors against depression and anxiety during an infection pandemic, frequent social interaction through videoconferencing and web-conferencing applications should be encouraged by the university authorities. This may help to maintain social interaction and integration with family members and friends, lowering the risk of depression and anxiety among university students.

## Conclusion

This study suggested that depression, anxiety, and stress have been highly prevalent among university students during the COVID-19 pandemic, even after the movement lockdown was lifted. It was found that COVID-19-related stressors, such as frustration from loss of daily routine and disruption of study, and clinical factors, such as having preexisting medical, depressive, and anxiety disorders, predisposed students to depression and anxiety. In contrast, family and friends social support and enrolling in medicine-related courses were associated with lower depression and anxiety. Steps to maintain a persistent flow of academic activities and social interaction may be necessary to curb depression and anxiety among university students in the context of the ongoing COVID-19 pandemic.

## Supporting information

S1 Appendix Socio-demographic and clinical factor questionnaire use in this study. (DOCX)
