# Supplementary material for: Depression, anxiety, and the COVID-19 pandemic: Severity of symptoms and associated factors among university students after the end of the movement lockdown
Source: PLoS One. 2021 May 27;16(5):e0252481. doi: 10.1371/journal.pone.0252481 (PMC8158968; doi:10.1371/journal.pone.0252481)
Supplement: S1 Appendix — (DOCX) [file pone.0252481.s001.docx]

**Socio-demographic and clinical factor questionnaire (Soal selidik sosio-demografi dan faktor klinikal)**

**Research no (No. penyelidikan):**

**Date (Tarikh):**

**Instruction: Please answer all the questions below.**

**(Arahan: Sila jawab semua soalan dibawah)**

(1) Umur (Age): tahun (years)

(2) Jantina (Gender):

Lelaki (Male) Perempuan (Female)

(3) Status perkahwinan (Marital status):

Berkahwin (Married) Belum berkahwin/janda/duda

(Single/divorcee/widow/widower)

(4) Adakah hubungan anda dengan suami/isteri terjejas semasa PKP? (Did you had any marital problem with your spouse during MCO?)

Ya (Yes) Tidak (No)

(5) Agama (Religion):

Islam (Muslim) Bukan Islam (Non-Muslim)

(6) Adakah mempraktikkan ajaran agama membantu anda menangani stres semasa PKP? (Did religion help you cope during the MCO and COVID-19?):

Ya (Yes)

Tidak (No)

(6) (a) Jika ya, sila nyatakan bagaimana mempraktikkan ajaran agama membantu anda menyesuaikan diri dengan perubahan hidup semasa PKP? (If yes, please state how religious belief helped you to adapt to the changes in life during MCO?)

(7) Jenis kursus yang diambil (Type of course undertaken):

Sains perubatan dan kesihatan (Medical science related) [BSc/MSc/PhD]

Sarjana muda dan sarjana perubatan (Medicine related)

[MBBS/MMed/subspeciality]

(8) Purata perbelanjaan bulanan (Average monthly expenses):

< RM 1000

RM 1000 – RM 3000

> RM 3000

(9) Anda tinggal dengan siapa semasa PKP dilaksanakan? (Who did you live with when MCO was enforced?):

Saya tinggal berseorangan (I live alone)

Saya tinggal bersama kawan-kawan atau rakan kursus (I live with friends or

course mates)

Saya tinggal dengan keluarga (I live with my family)

(10) Adakah anda risau tentang keluarga anda semasa PKP? (Were you worried about your family during the MCO?)

Ya (Yes)

Tidak (No)

(11) Adakah anda kecewa kerana kehilangan rutin harian semasa PKP? (Were you frustrated because of loss of daily routine during the MCO?):

Ya (Yes)

Tidak (No)

(11) (a) Jika ya, apakah rutin harian yang tidak dapat dibuat semasa PKP yang menyebabkan anda kecewa? (If yes, what were the daily routines which you were unable to do during MCO which cause your frustration?)

(12) Berapa jam anda menghadiri kelas dalam talian dalam seminggu semasa PKP? (How many hours did you attend online class in a week during MCO?

jam (hours)

(12) (a) Adakah anda rasa stres kerana pelajaran anda terganggu semasa PKP? (Did you feel stress because your study was disrupted during MCO?)

Ya (Yes)

Tidak (No)

(12) (b) Jika ya, apakah yang buat anda rasa stress apabila pelajaran anda terganggu semasa PKP? (If yes, what make you stress when your study was disrupted during MCO?)

(13) Sejarah penyakit medikal/surgikal (History of pre-existing medical/surgical illnesses):

Ya (Yes)

No (Tidak)

(13) (a) Jika ya, apakah penyakit medikal/surgikal yang anda hidap? (If yes, what is/are the medical/surgical illness which you have?)

(14) Sejarah penyakit psikiatri (History of pre-existing depressive and anxiety disorders):

Ya (Yes)

No (Tidak)

(14) (a) Jika ya, apakah penyakit psikiatri yang anda hidap? (If yes, what is/are the psychiatric illness which you have?)

(15) Adakah anda takut apabila anda mengalami gejala-gejala seperti batuk, demam dan selesema? (Were you afraid that you may have COVID-19 if you develop cough, flu or fever?):

Tidak (No)

Neutral (Neutral)

Ya (Yes)

(16) Adakah tempat tinggal anda semasa PKP merupakan lokasi yang mempunyai ramai kes jangkitan COVID-19? (Were COVID-19-positive cases prevalent in your area of living during the MCO?):

Ya (Yes)

Tidak (No)

(17) Adakah anda dikuarantin selama 14 hari semasa PKP kerana terdedah kepada kes positif COVID-19? (Were you quarantined for 14 days during MCO because of being exposed to COVID-19 positive cases?)

Ya (Yes)

Tidak (No)

Terima kasih kerana menjawab soalan (Thank you for answering the questions)
